# Ultrasonographic appearance of supraspinatus and biceps tendinopathy improves in dogs treated with low-intensity extracorporeal shock wave therapy: a retrospective study

**DOI:** 10.3389/fvets.2023.1238513

**Published:** 2023-08-07

**Authors:** Tari Kern, Jane Manfredi, Julia Tomlinson

**Affiliations:** ^1^Pawsitive Steps Rehabilitation and Sports Medicine, Rochester Hills, MI, United States; ^2^Pathobiology and Diagnostic Investigation, Michigan State University College of Veterinary Medicine, East Lansing, MI, United States; ^3^Twin Cities Animal Rehabilitation and Sports Medicine Clinic, Burnsville, MN, United States

**Keywords:** biceps, tendinopathy, supraspinatus, canine, shockwave, diagnostic ultrasound, piezowave, rehabilitation

## Abstract

**Objective:**

This study aimed to determine whether dogs with shoulder tendinopathy diagnosed via musculoskeletal ultrasound would show improvement in imaging after treatment using piezoelectric shockwave therapy and rest.

**Methods:**

Medical records were reviewed for dogs diagnosed with biceps and/or supraspinatus tendinopathy *via* musculoskeletal ultrasound, treated using piezowave shockwave, and re-imaged post-treatment. Data collected included patient signalment, duration and grade of lameness, prior rest, piezowave dose, and patient outcome, including a return to sport where applicable. Images were scored using an adapted ultrasound grading scale, in addition to obtaining cross-sectional area measurements. Statistics included Shapiro–Wilk tests (normality), Wilcoxon matched pairs signed rank tests (pre- vs. post-treatment comparisons), and Spearman's correlation coefficients (lameness grade vs. ultrasound score) (significant at *P* < 0.05).

**Results:**

In total, 26 of 30 dogs had pathology involving both the biceps and supraspinatus tendons in one limb, with 27 of 30 having tendon/s affected bilaterally. For both tendons, post-treatment cross-sectional area and ultrasound score were significantly lower than pre-treatment (*P* < 0.001). Lameness decreased clinically (*P* < 0.0001) following piezowave shockwave treatment regardless of the tendons involved, but the lameness score did not correlate with the ultrasound score for either tendon.

**Conclusion:**

Dogs with tendinopathy of the biceps brachii and supraspinatus showed significant improvement on follow-up musculoskeletal ultrasound and lameness evaluation after the treatment of their tendons using piezoelectric shockwave therapy with rest.

**Clinical significance:**

Canine biceps brachii and supraspinatus tendinopathy can cause variable lameness and ultrasonographic appearance, which improves after shockwave therapy and rest. The ultrasound scoring system and cross-sectional area assessment provide useful outcome measures for clinical cases.

## 1. Introduction

Shoulder tendinopathy in dogs has been reported to be associated with lameness, including pathology of the biceps tendon of origin (1) and the supraspinatus (2, 3). Biceps and supraspinatus tendinopathies have been recognized to be secondary to concurrent shoulder degenerative joint disease and/or elbow pathology, but many cases have lacked concurrent pathologies (4). Historically, diagnosis of these tendinopathies has been determined via a combination of physical examination, radiographs, ultrasonography and MRI. Advanced diagnostic imaging provides more information about the structural tissue changes in the lame limb and has often identified concurrent tendinopathy in the contralateral shoulder, which was free from clinical signs (4).

The use of musculoskeletal ultrasound in veterinary medicine allows for better characterization of the changes affecting musculotendinous structures and tendons since normal characteristics of the canine shoulder structures have been well documented (5–10). Tendon pathology noted on musculoskeletal ultrasound includes abnormal fiber pattern, tendon enlargement, variable echogenicity within the tendon, bone modeling at the tendon insertion, mineralization, and increased amount of fluid within the tendon sheath (7–12). Mineralization and calcification may also be present in shoulder tendinopathy cases, either within the muscles or within the tendons, or both. However, their clinical significance is not well defined as some dogs may be clinically normal, regardless of the presence of mineralization (2). Other studies have documented that mineralizations are likely to recur even following surgical excision (13). Additionally, dogs with chronic changes identified in shoulder tendons *via* ultrasound may not appear to be clinically affected (14). Few studies have assessed musculoskeletal ultrasound findings before and after treatments for canine shoulder tendinopathy with the intention of evaluating the relationship between ultrasonographic pathology and clinical performance (15, 16). Musculoskeletal ultrasound has been used extensively as a diagnostic tool in horses for evaluating lameness, diagnosing tendinopathy, and also following clinical response to treatment therapies; however, there are no studies correlating ultrasound score with lameness or healing in dogs (17, 18).

There are several treatment options for supraspinatus and biceps tendinopathy in dogs. Historically, conservative management of shoulder tendinopathy was predominantly directed at restricted activity and reducing repetitive trauma. The use of non-steroidal anti-inflammatory drugs (NSAIDs) did not show significant clinical improvement, with over 74% of the dogs failing to respond (11). Rehabilitation therapy techniques including massage, manual therapies, therapeutic ultrasound, and laser therapy have also been used with mixed results (11, 19). Surgical options for the treatment of tendinopathies have demonstrated variable success (3, 20). The use of stem cell therapy, using adipose-derived cells and bone marrow aspirate concentrate, in conjunction with platelet-rich plasma, displayed supraspinatus tendon fiber improvement with musculoskeletal ultrasound assessment pre- and post-treatment (15, 16). Treatment of shoulder tendinopathy in dogs using extracorporeal shockwave has been validated as a therapeutic option (4, 21). However, not all of the dogs in these studies were imaged with diagnostic ultrasound before therapy and none of them were imaged after therapy, instead relying on client interviews for follow-up information (4, 21).

Shock waves are fast, high-pressure impulses (5–120 MPa in ~5 ns), followed by a low tensile negative pressure impulse (22). The mechanism of action for therapeutic shockwave is defined as mechanical transduction, where the movement of tissue stimulates biological processes (23). Shock waves enhance fibroblast proliferation *in vitro* (24). Additionally, shock waves have anti-inflammatory effects, including modulation of nitrous oxide (25, 26), stimulation of neovascularization (27), production of lubricin (28), and production of growth factors, such as IGF, TGF-β, and VEGF (24, 29).

The piezoelectric form of shockwave has only recently been used in veterinary medicine but has been used for human orthopedic conditions (30). The piezoelectric technique involves exciting a large number of piezo crystals by a rapid electrical discharge which causes a pressure change inside the probe head, generating a pulse. The arrangements of the crystals cause self-focusing of the waves toward the target center. The focal area is smaller than the other types of shockwave units, and a piezowave is often described as a low-intensity focused shockwave (31–33). Currently, only one case report utilizing piezoelectric shockwave for the treatment of canine supraspinatus tendinopathy has been published (34). The human research literature similarly has very few publications evaluating piezoelectric shockwave for tendinopathy treatment (30, 35–37). Anecdotally, piezoelectric shockwave has been used over the last decade in veterinary medicine for a myriad of conditions with apparently good clinical results. However, clinical research documentation of tissue response to treatment is lacking. We hypothesized that dogs with tendinopathy of the biceps brachii and supraspinatus as diagnosed *via* musculoskeletal ultrasound would show improvement on follow-up musculoskeletal ultrasound evaluation after treatment of their tendons using piezoelectric shockwave therapy combined with rest. Additionally, we hypothesized that improvements in ultrasound scores following treatment would translate into improvements in the lameness scores for the dogs in the study.

## 2. Materials and methods

### 2.1. Case selection

A retrospective review of all canine patient medical records from one private small animal rehabilitation practice was performed to identify cases receiving piezoelectric shockwave therapy for tendinopathy between January 2016 and September 2021. Because this was a retrospective study that retrieved data from medical records without identifying information, this study was exempted from needing Institutional Animal Care and Use Committee review and approval. Inclusion criteria for the study required a diagnosis of supraspinatus and/or biceps tendinopathy *via* musculoskeletal ultrasound following presentation for the evaluation of lameness in one forelimb with signs of pain appreciated upon manipulation of the shoulder joint of the lame limb. Additionally, cases included in this study needed to have completed a series of piezoelectric shockwave treatments performed once weekly for 3 weeks, followed by a recheck evaluation and post-treatment musculoskeletal ultrasound performed 3 weeks after the third piezoelectric shockwave treatment. Follow-up beyond the last treatment was required to be a minimum of 3 months.

Cases were excluded from the study if dogs were diagnosed with concurrent or later medial shoulder instability (MSI), laxity with pain on abduction associated with medial shoulder syndrome (MSS), or the presence of elbow pain and pathology (pain or radiographic disease at the time of initial evaluation).

Shoulder instability or laxity was determined under sedation at the time of imaging with diagnostic ultrasound and at reimaging 3 weeks after shockwave therapy. The determination was made by assessing for pain on abduction while awake and by finding excess motion on passive abduction of the joint in full extension while sedated. Gross instability identified on physical examination was also evaluated radiographically, looking for a lack of congruency of joint surfaces. Laxity in the absence of gross instability was defined as an abduction angle ≥10 degrees more than the contralateral limb or ≥45 degrees (38). Dogs were also excluded from the study if the medical records, including the presence of ultrasound images to review, were incomplete.

### 2.2. Medical record review

Information gathered from the medical records included patient signalment, duration of lameness, lameness score pre- and post-treatment, diagnosis, treatment, and outcome, including a return to sport where applicable. Written reports from the diagnostic ultrasound were not reviewed as the images were scored as part of the study. Lameness was graded using a scale (0–5) where 0 is no lameness, 1 represents slight lameness in response to exercise, 2 represents slight lameness without exercise, 3 indicates moderate lameness, 4 represents severe lameness, and 5 indicates non-weight-bearing lameness (39).

### 2.3. Ultrasound evaluation and scoring

All but three dogs were bilaterally ultrasonographically imaged prior to treatment, and those unilaterally imaged were due to owner preference. The clinical protocol for obtaining an image series was to scan each tendon from its origin to the musculotendinous junction using a 16 MHz linear transducer *via* multiple cineloops of the entire tendon in cross section and longitudinal planes. Single frame capture was used to measure the cross-section. Cross-sectional and longitudinal images were taken after clipping the hair and applying coupling gel according to convention (40). Images were reviewed by one author (JT) on the machine used for patient imaging (GE NextGen LOGIQ e, GE Healthcare, USA).

The ultrasonographic appearance of each of the affected tendons treated was scored retrospectively using a scale developed by one author (JT), adapted from a previously published human medical scoring system (41) (Table 1). The score ranged from 0 (normal tendon) to 15. Scoring was performed using previously recorded ultrasound images (static and video clips) of each patient pre-treatment (PRE) and post-treatment (POST). Written patient reports were not reviewed by this author (JT) at the time of scoring, and the scoring order was randomized for each patient (PRE or POST could be scored first). The digital images and video clips were reviewed and assessed with respect to the following criteria: reduced echogenicity, fiber orientation, length of total tendon affected, fiber definition in the affected region, fluid in the biceps sheath, mineral in the tendon, and adhesion to other structures for the supraspinatus and biceps brachii tendons (Table 1).

**Table 1:** Ultrasound scoring system adapted for canine use (41).

**Amount of reduced echogenicity in the affected region**	**Grading range**	**Grade criteria**
	0	Normal echogenicity
	1	Hypoechoic < 30% of cross section
	2	Hypoechoic 30–50% of cross section
	3	Hypoechoic >50% of cross section
Fiber orientation in the affected region	**Grading range**	**Grade criteria**
	0	Normal
	1	Disorganization of < 30% of visible fibers
	2	Disorganization of 30–50% of visible fibers
	3	Disorganization of >50% of visible fibers
Length affected (total)	**Grading range**	**Grade criteria**
	0	Normal
	1	< 30% of tendon length
	2	30–50% of tendon length
	3	>50% of tendon length
Fiber definition in the affected region	**Grading range**	**Grade criteria**
	0	Normal
	1	Poor to no definition of < 30% of visible fibers
	2	Poor to no definition of 30–50% of visible fibers
	3	Poor to no definition of >50% of cross section
Fluid in biceps sheath	**Grading range**	**Grade criteria**
	0	No excess fluid
	1	Excess fluid
Mineral in tendon	**Grading range**	**Grade criteria**
	0	No mineral
	1	Mineral present within the tendon
Adhesion to other structure	**Grading range**	**Grade criteria**
	0	No adhesion/normal glide
	1	Adhesion affecting tendon glide

The composite score (0–15) is made from each category with normal = 0.

In addition to scoring their appearance, the cross-sectional area of each tendon (cm^2^) as measured and recorded on the images reviewed was collected PRE and POST. The cross-sectional area of the biceps tendon is taken routinely at the level of the glenoid cavity, the proximal biceps groove (where the groove is easily visible), and just proximal to the musculotendinous junction (before any muscle fibers are seen). However, for the purpose of this study, the cross-sectional area was measured in the center of the affected length (which was noted for shockwave parameters). For the supraspinatus, the cross-sectional area was taken in the center (proximal to distal) of the insertional region where fibrocartilage is visible, and this was always confirmed to be orthogonal to the longitudinal view, ensuring no obliquity. For supraspinatus findings, this insertional region was affected in all dogs with supraspinatus pathology; generally, the distal 1.5–2 cm of the tendon was affected in cases with pathology.

### 2.4. Treatment parameters

All dogs were treated using a piezowave-focused shockwave (Piezowave2-VET Richard Wolf—ELvation Medical GmbH) once weekly for three treatment sessions. Treatment was performed within 10 days of initial imaging; therefore, the hair remained clipped short during treatment. Patients were treated in lateral recumbency but were un-sedated. The focused penetration depth was determined at the time of imaging, measuring the distance from the skin to the center of the tendon and the appropriate gel pad (5 or 10 mm) for the depth of treatment used. The site in each tendon treated and the length of the treatment zone were selected based on the affected tendon length plus an additional 0.5 cm buffer length proximal and distal to the affected region as imaged on diagnostic ultrasound. The treatment prescription included distance from or to bony landmarks to aid in correct placement of the probe. If ultrasound evaluation identified pathology in both limbs, both limbs and all affected tendons were treated, regardless of the lameness laterality. The biceps brachii tendons were treated with 500 pulses per site, at a frequency of 6 pps (pulses per second), with each site defined as a 1 cm treatment area along the affected length of the tendon. The supraspinatus tendon was treated similarly, but 750 pulses were administered per site according to the clinically defined protocols based on the manufacturer's recommendations. The energy flux density (EFD in mj/mm^2^) was prescribed within a range and applied at the highest end of the range that the patient would tolerate. The total EFD range used per tendon in the study population was 0.117–0.173 mj/mm^2^ (mean ± SD 0.15 ± 0.03). All dogs included in the study were treated only with piezoelectric shockwave, and exercise restrictions were in place, which included leashed walks for elimination and prevention of running, jumping, and climbing stairs for the duration of their treatment until repeat musculoskeletal ultrasound.

### 2.5. Statistical analysis

Descriptive statistics were used for age at presentation, lameness score, and lameness duration (39). Data were analyzed using the statistical program Prism 8 (Graph Pad Software, San Diego, CA, USA). For the lameness score, the cross-sectional area data, and the ultrasound score data with each tendon, Shapiro–Wilk tests were used to assess data for normality, and a Wilcoxon matched pairs signed-ranked test was used to assess differences between PRE and POST as data were not normally distributed. Spearman's correlation coefficient was used to assess the correlations between lameness grade and ultrasound score. The significance level was set as *P* < 0.05.

## 3. Results

In total, 4,619 medical records were reviewed; 41 dogs had lesions of the biceps brachii and/or supraspinatus, and 30 dogs met all of the inclusion criteria. The other 11 dogs were found to have concurrent medial shoulder pathology and/or elbow problems, excluding them from the study.

### 3.1. Signalment

The study population consisted of 30 dogs, of which 16 were males (5 intact and 11 neutered) and 14 were females (3 intact and 11 spayed). For those 30 dogs, the mean ± standard deviation (SD) age was 5.18 ± 2.12 years, with a body weight of 28.46 ± 15.42 kg (62.43 ± 33.94 lbs.). Six of the 30 dogs were under 18.2 kg (40 lbs.).

There were 24 cases in pure bred dogs, including 9 Labrador Retrievers, 3 German Shepherds, 2 Border Collies, 1 Golden Retriever, 1 English Springer Spaniel, 1 Brittany Spaniel, 1 Rottweiler, 1 Mastiff, 1 Chesapeake Bay Retriever, 1 Flat-coated Retriever, 1 Pyrenean Shepherd, 1 Miniature Bull Terrier, and 1 Munsterlander. Additionally, six mixed-breed dogs were within the study population.

In total, 21 dogs were athletes or working dogs. Many of the athletes participated in one or more sports, including 12 agility dogs, 10 hunting/hunt trial dogs, 2 obedience dogs, and 1 dock diving dog. Of the working dogs, one was a canine officer and one was a search and rescue-trained dog; nine were solely pet dogs.

### 3.2. Previous treatment

All but seven dogs had periods of rest and treatment using NSAIDs prior to referral for ongoing issues. The duration of rest varied from 2 weeks to 2 months for 12 of these dogs, who continued to be lame despite rest. A reduction in lameness but not resolution, during rest, was reported for six dogs. Some of these dogs also had multiple periods of rest prior to referral. However, it was reported that all of the dogs previously treated with periods of rest had worsening or recurrence of lameness occurred once normal activities, such as running or jumping, were allowed again. In the other seven cases, it was unknown whether the dogs were rested prior to referral for evaluation.

### 3.3. Lameness

All dogs had lameness on presentation; 13 dogs had right forelimb lameness and 17 dogs had left forelimb lameness. The duration of lameness in the study population prior to presentation ranged from 2 to 104 weeks with a mean ± SD of 26.14 ± 27.31 weeks. Lameness was scored as median [interquartile range (IQR)] of 2 (2–3) PRE and 0 (0–0.25) POST. Lameness decreased clinically and significantly (*P* < 0.0001) following piezowave shockwave treatment for injury to both the biceps brachii and supraspinatus tendons.

### 3.4. Tendons affected

At presentation, tendons were affected in both front limbs in all but three cases upon musculoskeletal ultrasound evaluation. Of the 57 biceps and 57 supraspinatus tendons reviewed, there were 54 affected biceps tendons and 44 affected supraspinatus tendons. In total, 18 dogs had both biceps and supraspinatus tendinopathy bilaterally affected, whereas 8 dogs had only one limb with biceps and supraspinatus tendinopathy, and the other limb had only the biceps affected. Four dogs had biceps pathology and no discernible supraspinatus pathology in either limb, and none had supraspinatus pathology in the absence of biceps pathology. If ultrasound evaluation identified pathology in both limbs, both limbs and all affected tendons were treated, regardless of the lameness laterality.

### 3.5. Ultrasonographic appearance and scoring

#### 3.5.1. Cross-sectional area

**Figure 1:**
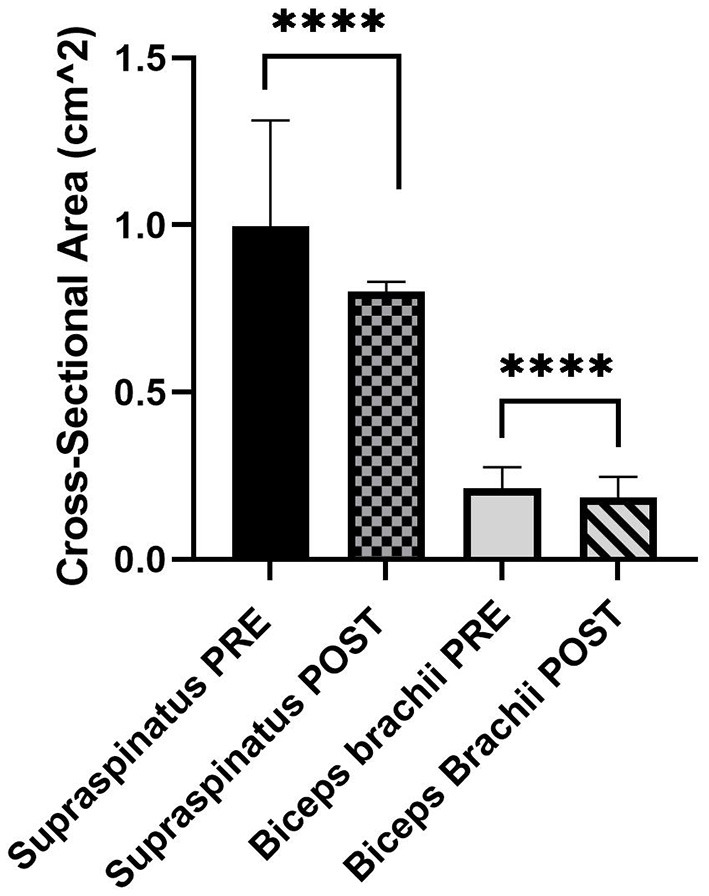
Median and 95% confidence interval (CI) of PRE and POST cross-sectional area (cm^2^) of the supraspinatus (*n* = 44) and biceps brachii tendons (*n* = 54) (*N* = 30 dogs). ****P < 0.0001.

Data from both biceps brachii and supraspinatus tendons (PRE and POST) were not normally distributed. The cross-sectional area for supraspinatus was calculated as a median [interquartile range (IQR)] of 0.95 (0.76–1.23) cm^2^ PRE and 0.80 (0.68–0.90) cm^2^ POST. The cross-sectional area for biceps brachii was calculated as a median (IQR) of 0.20 (0.18–0.24) cm^2^ PRE and 0.18 (0.15–0.20) cm^2^ POST. For both tendons, the POST cross-sectional area was significantly lower than PRE (Figure 1, *P* < 0.0001).

#### 3.5.2. Ultrasound score

**Figure 2:**
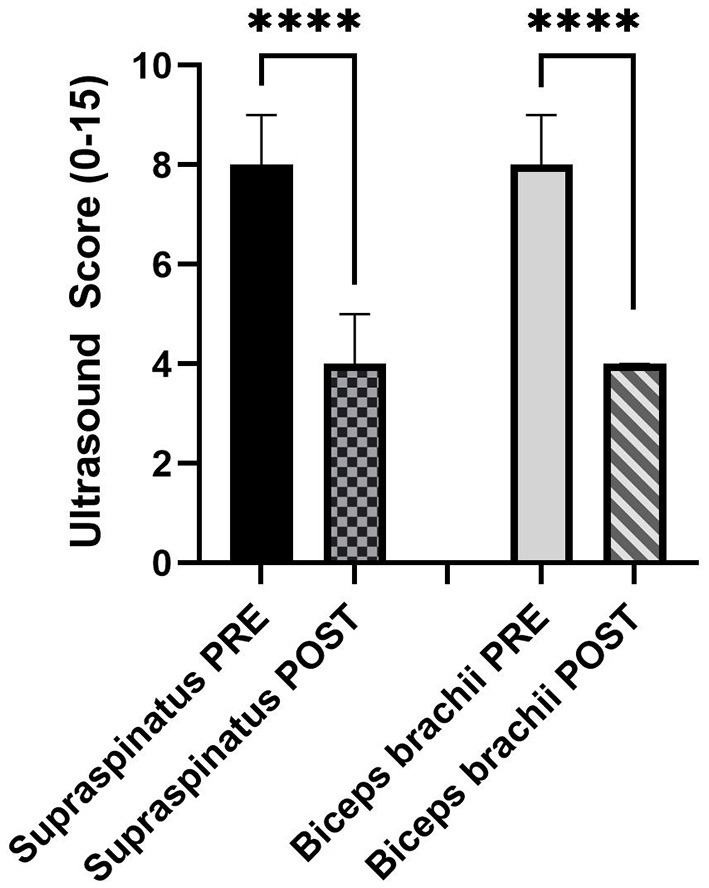
Median and 95% confidence interval (CI) of PRE and POST ultrasound score (0–15) of the supraspinatus (*n* = 44) and biceps brachii tendons (*n* = 54) (*N* = 30 dogs). ****P < 0.0001.

The ultrasound score for the supraspinatus was a median (IQR) of 8 (6–9) PRE and 4 (0.25–6) POST. The ultrasound score for the biceps brachii was a median (IQR) of 8.5 (6.75–9) PRE and 4 (2–5) POST. A Wilcoxon-matched paired signed-rank test was used due to the non-normality of the data. For both tendons, POST ultrasound scores were significantly lower (both *P* < 0.0001; Figure 2).

**Figure 3:**
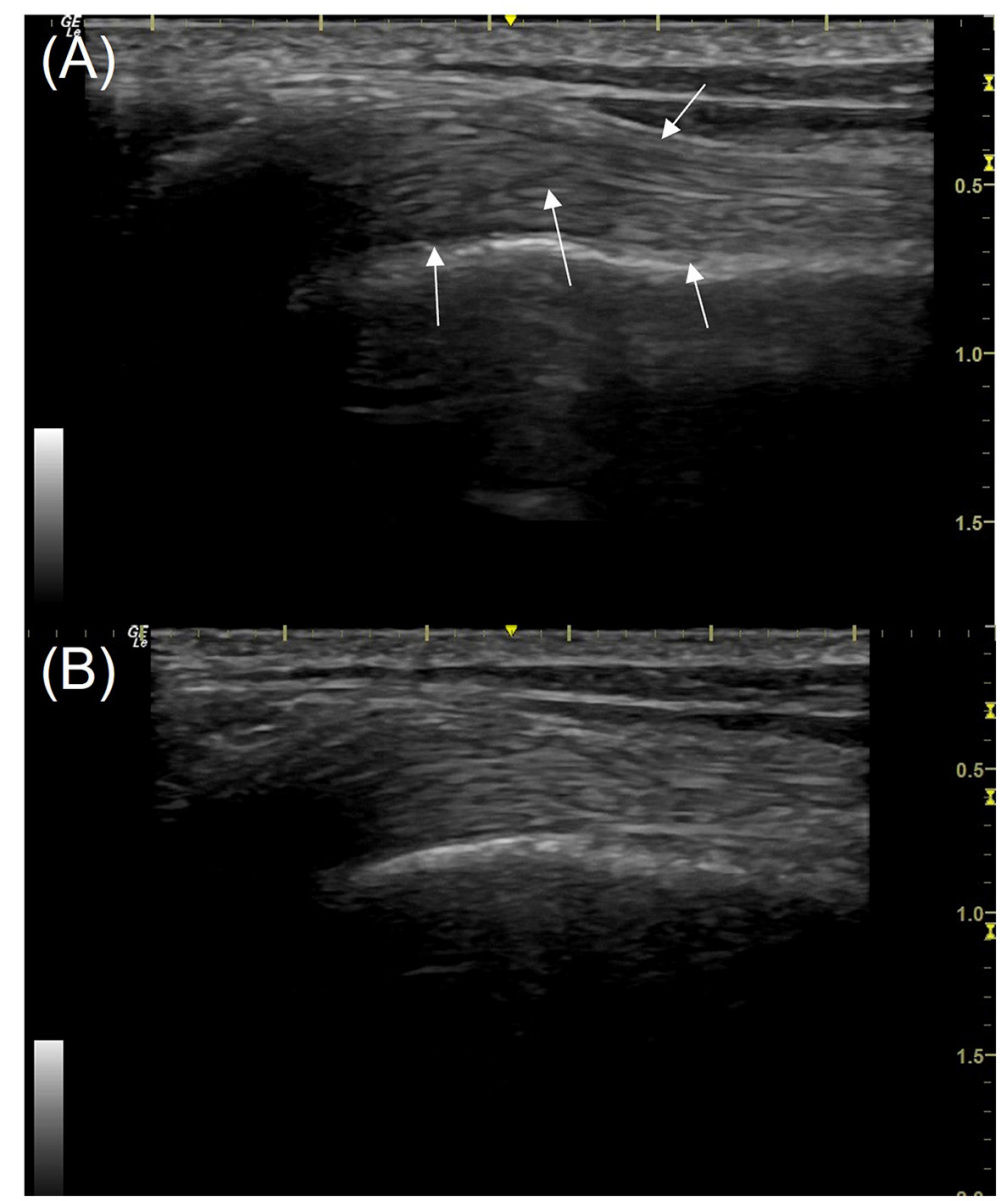
Longitudinal view of the mid biceps tendon (the right limb) from a patient in the study; images taken using a 16 MHz linear transducer. **(A)** Pre-treatment with piezowave therapy, **(B)** 3 weeks post-treatment. The ultrasound score of the tendon pre-treatment is 6 with multiple hypoechoic regions (white arrows). The score post-treatment **(B)** is 3, some areas of reduced echogenicity remain but fibers are more clearly defined. The proximal is to the left in this image.

**Figure 4:**
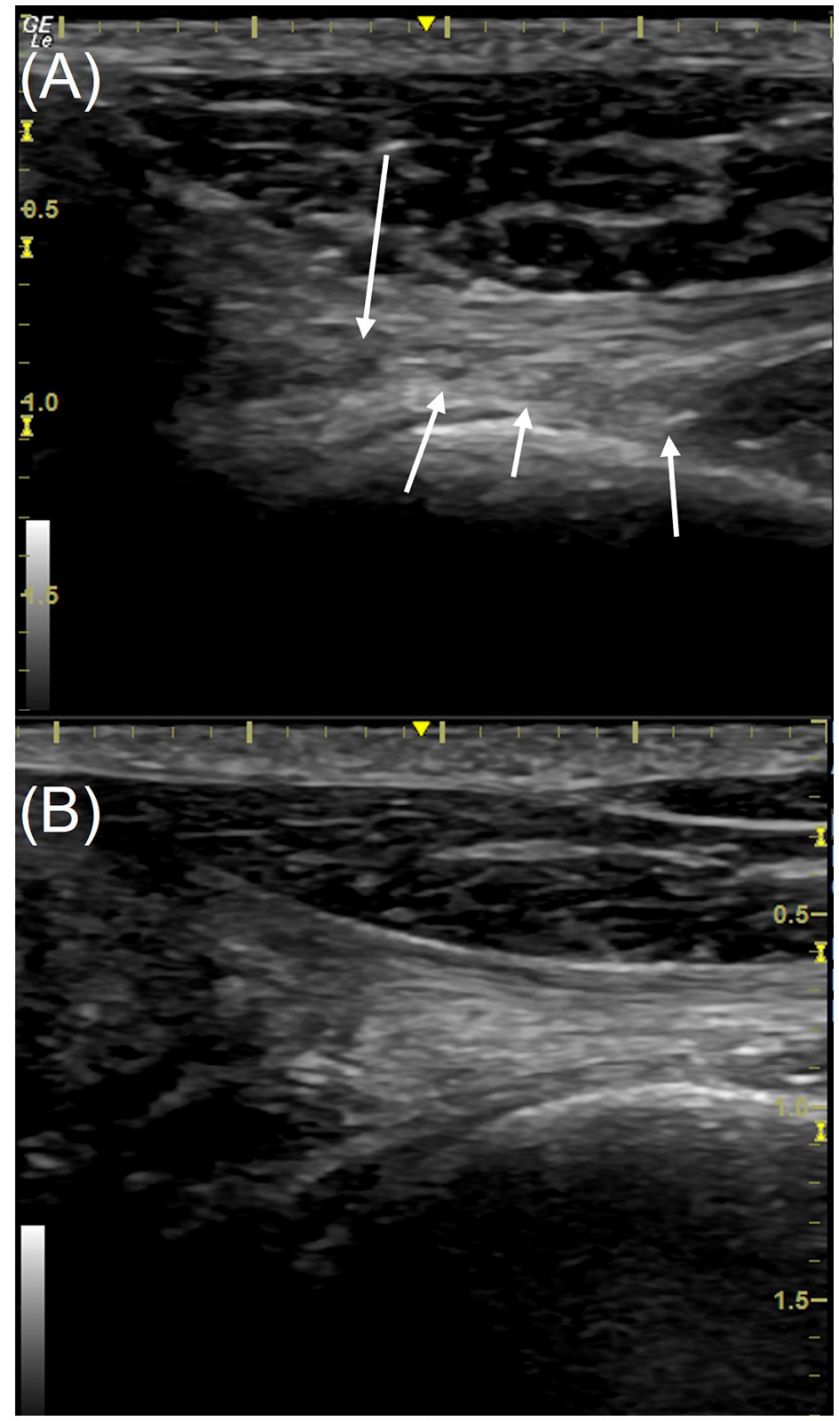
Longitudinal view of the distal supraspinatus tendon (left limb) from a patient in the study, the anechoic insertional fibrocartilage is on the left of each image. Images are taken using a 16 MHz linear transducer. **(A)** Pre-treatment with piezowave therapy, **(B)** 3 weeks post-treatment. The ultrasound score of the tendon pre-treatment **(A)** is 9 with multiple hypoechoic regions (white arrows), in addition to the loss of fiber definition and orientation. The score post-treatment **(B)** is 5, some areas of reduced echogenicity remain but fibers are more clearly defined and better oriented; less overall length of the tendon is affected. The cranial is to the left in this image.

The sonographic findings included reduced echogenicity and reduced fiber definition and fiber disorganization (Figures 3, 4). Mineralization was found in 14 biceps tendons and 19 supraspinatus tendons PRE, all of which had other concurrent pathology within the tendon. POST mineralization was not identified in 10 out of 14 biceps tendons and 4 out of 19 supraspinatus tendons. Post-treatment mineralization was present in 4/14 biceps and 15/19 supraspinatus tendons.

### 3.6. Relationship between ultrasound score and lameness grade

**Figure 5:**
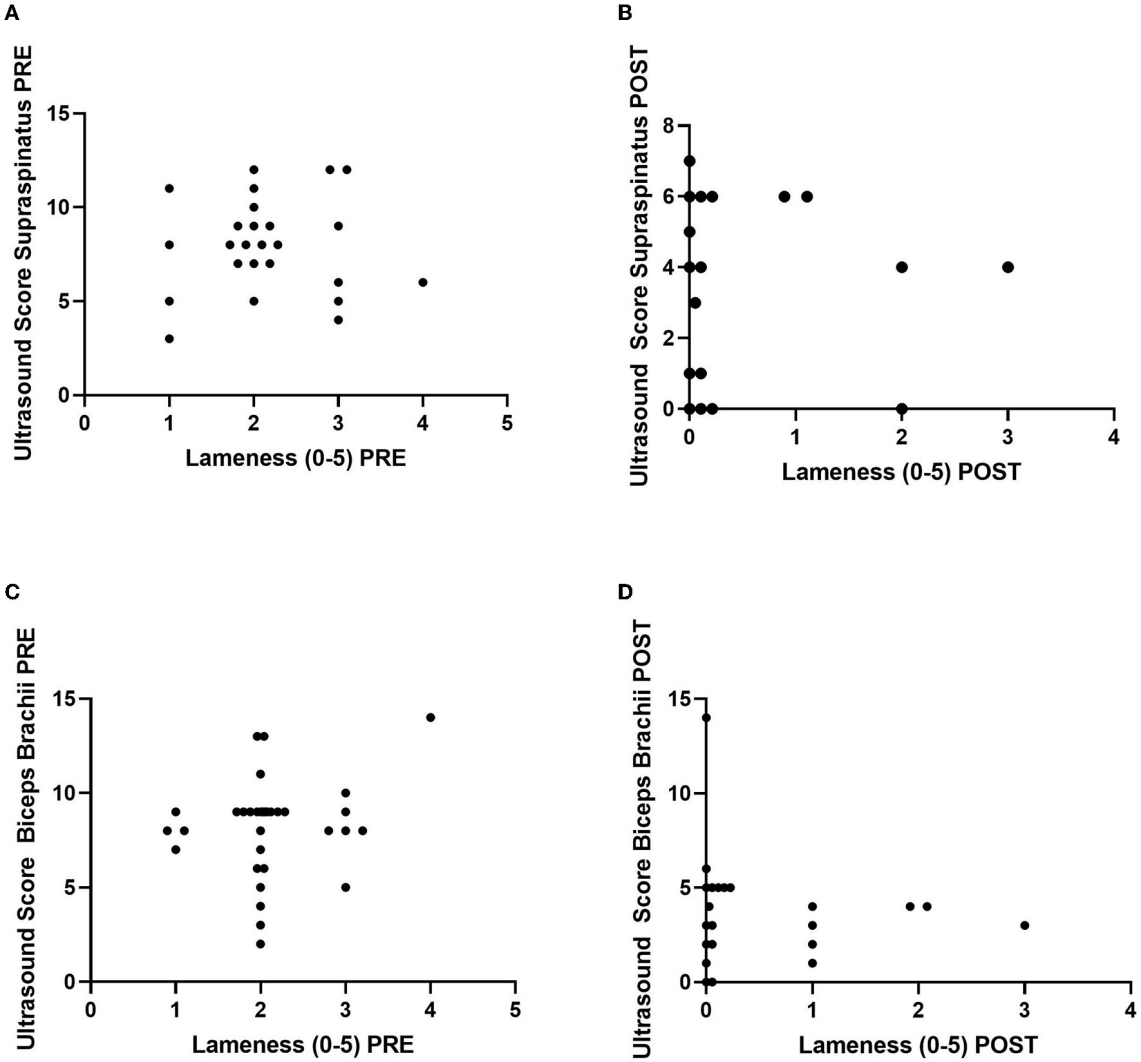
Correlations between lameness grade (0–5) vs. ultrasound score (0–15) of the supraspinatus (*n* = 25 dogs/tendons) **(A)** PRE and **(B)** POST (top) and the biceps brachii (*n* = 30 dogs/tendons) **(C)** PRE and **(D)** POST (bottom). Ultrasound score from the lame leg only is graphed. Five dogs do not have supraspinatus tendon injuries.

There was no correlation between lameness and ultrasound score at either the PRE or POST timepoint for the tendons involved (Figure 5). The ultrasound score for the supraspinatus PRE vs. lameness had a Spearman's *rho* of 0.02 (*p* = 0.92), while POST had a Spearman's *rho* of 0.09 (*p* = 0.67). The ultrasound score for the biceps brachii PRE vs. lameness had a Spearman's *rho* of 0.08 (*p* = 0.66), while POST had a Spearman's *rho* of 0.18 (*p* = 0.34).

### 3.7. Follow-up

The cases within this study were followed for between 3 and 12 months (mean ± SD was 8.7 ± 3.7 months) after their shockwave treatment. The case information was available for all 30 dogs at 3 months post-treatment. The majority of the dogs in this study were able to return to their normal activities without lameness.

In total, 18 of the canine athletes/working dogs (85.7%) were able to return to their sport/work by 3 months following treatment; however, one of these dogs was euthanized 3 months later due to an unrelated problem. Three dogs did not return to sport. One dog developed neck pain and rear limb ataxia. One (a German Shepherd) dog developed a gracilis strain and subsequent contracture 2 months after forelimb therapy concluded. One dog had persistent shoulder regional pain that did not respond to intraarticular injection of triamcinolone and hyaluronic acid, and the owner declined further diagnostics. As of 6 months following treatment, 18 out of 21 of the canine athletes continued to have no forelimb lameness recurrence. Medical record information was available for 11 of the 21 athletes/working dogs at 12 months after treatment, which confirmed that these dogs also had no lameness observed.

Eight of the nine pet dogs returned to normal activity (88.9%) by 3 months post-treatment. One dog developed a carpal sprain 3 months after the last piezowave treatment (this dog persistently jumped down from high surfaces in the house) but subsequently recovered and returned to normal activity. Follow-up medical record information was available for seven pet dogs at 6 months after treatment and five pet dogs at 12 months post-treatment, revealing no lameness recurrence at either period.

## 4. Discussion

The hypothesis that the ultrasonographic appearance of biceps and supraspinatus tendinopathy in dogs would improve after piezowave shockwave therapy and rest was accepted. The study population had variable degrees of typical ultrasonographic tendinopathy signs prior to treatment with shockwave. Following piezowave treatment, significant improvements were noted on ultrasound evaluation, including reduced echogenicity, improved fiber definition, and better fiber organization.

This study also confirmed several previously noted clinical patterns for dogs with shoulder tendinopathy. Tendinopathies of the biceps brachii and supraspinatus have been reported to be most commonly seen in medium-to-large breed dogs and also in highly active dogs (1, 42). Most of the patients (80% or 24 out of 30 dogs) featured in this study weighed over 18.2 kg (40 lbs.), with 20% (6 out of 30 dogs) being small breed dogs, weighing < 18.2 kg. Additionally, 70% of the studied dogs (21 out of 30 dogs) had been actively engaged in canine sports or other canine work, while 30% (9 out of 30 dogs) were pets without sporting activities. It should be noted that the general population base for the practice in which the study was conducted is comprised of ~45% canine athletes, and 47% (14 of 30) of the study group were hunting breeds.

The lameness score was subjective in these cases, using a commonly utilized lameness scale (39). Scores were lower POST as compared with PRE, with 23 of 30 dogs having no lameness POST.

While it appears that piezowave shockwave therapy resulted in improved lameness in our study population, it should be noted that there was a concurrent rest period of 6 weeks with the treatment phase. The relative contribution of these factors cannot be determined in this study. Additional investigation using a prospective placebo-controlled study with a control group of dogs only rested, as detailed in the limitations section below, would be required to address this question.

When considering a treatment modality, it is important to compare its effectiveness with other therapy options in order to assess the pros and cons of each. This study provided some initial information on the efficacy of piezowave shockwave therapy as a treatment for tendinopathy of the biceps and supraspinatus in dogs. A study evaluating the response to surgical treatment of non-mineralized supraspinatus tendinopathies in dogs found recovery to be very good in 15 out of 27 dogs, good in 8 out of 27, and sufficient in 4 out of 27 dogs (3). The piezowave shockwave treatment protocol used in this study demonstrated significant improvement in the ultrasound scores in the majority of dogs, without the need for anesthesia or an invasive approach.

Most prior studies using shockwave therapy for tendinopathy treatment have lacked measurable outcome parameters following treatment to assess healing within the tissues affected. Instead, subjective evaluations, such as lameness assessment, clinical performance, or a client outcome assessment, have been used. Advances in diagnostic imaging have shown that abnormalities may still be present in dogs that may appear clinically normal. Ultrasound has been determined to be an accurate tool in the assessment of tendon pathology. Abnormal ultrasound findings are significant in that they may indicate pathology that results in current lameness, or they may indicate a pathology that will be expressed later as clinical lameness and be associated with signs of pain. This study used an ultrasound scoring method in an attempt to reduce subjectivity. While clinical improvement is important, an improved imaging score may provide additional information on the degree of recovery and eventually aid in providing better prognosis and assist in planning a return to normal use for the patient.

This ultrasound scoring method took into account both the affected length of the tendon imaged and the proportion of abnormal appearing tissue within that affected length. The cross-sectional area was also measured because, in cases of tendinopathy, the tendon is usually enlarged (43, 44), and therefore, a reduction in size indicates a positive response to treatment. Comprehensive written reports are conventionally made following musculoskeletal ultrasound imaging, and while descriptors of sonographic findings can be helpful in addition to image storage and review, a scoring system has the potential to improve interobserver reliability and intraobserver reliability between different time points. The scoring system was adapted from a previous study, where a similar scoring system was validated using a comparison between a musculoskeletal radiologist and a non-radiologist after a training period for the non-radiologist; good reliability was found for every tendon parameter except mineralization (41). In this study, one experienced ultrasonographer (JT) evaluated the tendons; therefore, mineralization was added as a part of the composite score, but the size and the number of mineral deposits were not detailed, just the presence or absence of minerals. Not quantifying mineralization does affect the ability to assess for reduction of mineralization; however, it is challenging to quantify mineralization when there may be multiple pinpoint areas. Therefore, it was determined to simply acknowledge the presence or absence of minerals.

Examining the lameness assessment for the patients within this study, there was no statistically significant correlation found between lameness scores and ultrasound scores. The same is frequently found in human medicine (41). There is also a reported lack of correlation between the radiographic severity of osteoarthritis and the degree of lameness (45, 46). This is best explained by the fact that many dogs are clinically normal with respect to shoulder tendinopathy and may not demonstrate lameness at all. It should be noted that within our patient population, several dogs actually had worse ultrasound scores on their “unaffected” non-lame side. A prior study noted the presence of bilateral tendinopathy in dogs despite unilateral lameness (11), which was consistent with our findings, where 90% of the dogs (27 out of 30 dogs) in this study had bilateral pathology confirmed *via* musculoskeletal ultrasound at presentation. As a result, using a lameness assessment alone as a measure for tendon healing is not recommended. The use of additional, more specific outcome measures, such as musculoskeletal ultrasound reassessment and scoring, should be encouraged, with the goal of reducing the risk of tendon rupture and injury recurrence.

The limitations of this study include its retrospective design, small study group, possible inconsistency in ultrasound measurements, and potential risk of bias. There was no control group in the study, which may lead some to suggest that improvements noted in the tendon might be related to rest alone. However, most of our patient population had already been treated with at least one cycle of rest and medical management prior to referral, without resolution of their clinical signs. Additionally, prior veterinary studies have reported failure of conservative management treatments, including rest, NSAID use, and rehabilitation therapy (3, 11, 47). Recent studies in human medicine indicate that rest does not improve tendinopathy, but that a slowly controlled progressive loading program combined with other pain-relieving treatment modalities may be beneficial in the successful management of tendinopathy cases (41). The dogs in this study were restricted in activity during the shockwave treatment period and for 3 weeks after, pending reimaging. No strengthening exercises or specific medications were prescribed. Following shockwave treatment, the majority of dogs treated had both improved ultrasound scores for their shoulder tendons and significant improvements in lameness. In fact, forelimb lameness was resolved in most dogs, even 6 months following treatment. Though the authors cannot state that the reduction in ultrasound score and cross-sectional area of affected tendons was only due to the piezowave shockwave therapy, the absence of concurrent treatments other than rest, and the fact that 23 of the 30 dogs had already undergone a period of rest and had not improved clinically, does indicate that the shockwave modality combined with rest does show promise vs. rest alone. The development of a prospective study using shockwave therapy, coupled with a slowly controlled progressive loading exercise program and tracked with measurable assessment outcomes, such as musculoskeletal ultrasound scoring and kinematic evaluation parameters, would be an ideal next step in studying canine shoulder tendinopathy treatment success.

## 5. Conclusion

Musculoskeletal ultrasound has proven beneficial in diagnosing shoulder tendinopathy in dogs but can also be helpful in monitoring the overall success of treatments used. This retrospective study was the first to track canine shoulder tendinopathy patient outcomes following piezoelectric shockwave therapy using musculoskeletal ultrasound *via* an adapted tendon ultrasound grading scale. Dogs diagnosed with tendinopathy of the biceps brachii and supraspinatus using musculoskeletal ultrasound showed significant improvement in follow-up musculoskeletal ultrasound evaluation after treatment of their tendons using piezoelectric shockwave therapy.

## Data availability statement

The original contributions presented in the study are included in the article/supplementary material, further inquiries can be directed to the corresponding author.

## Ethics statement

Ethical review and approval was not required for the animal study because this retrospective study was performed in clinical practice. Written informed consent for participation was not obtained from the owners because this was a retrospective study where animals were not uniquely identified.

## Author contributions

TK and JT contributed to the study design, data collection, and analysis. JT reviewed and scored ultrasound images. JM performed the statistical analyses. JT, TK, and JM contributed to writing the manuscript. All authors contributed to the manuscript revision, read, and approved the submitted version.
